# Anti-inflammatory effect of combined tetramethylpyrazine, resveratrol and curcumin in vivo

**DOI:** 10.1186/s12906-017-1739-7

**Published:** 2017-04-27

**Authors:** Long Chen, Tianjun Liu, Qiangsong Wang, Juan Liu

**Affiliations:** Tianjin Key Laboratory of Biomedical Materials, Institute of Biomedical Engineering, Chinese Academy of Medical Sciences & Peking Union Medical College, Tianjin, 300192 China

**Keywords:** Tetramethylpyrazine, Resveratrol, Anti-inflammatory, Curcumin, Uniform design, Acute and chronic inflammation, Toxic study

## Abstract

**Background:**

Resveratrol and curcumin, as natural flavones products, have good therapeutic effect in acute and chronic inflammation; on the other hand, tetramethylpyrazine (TMP) has angiogenesis and vessel protection effect as well as anti-inflammatory function. In this paper, the anti-inflammatory effect of the tetramethylpyrazine, resveratrol and curcumin (TRC) combination in acute and chronic inflammation was reported in vivo.

**Methods:**

The dose of the combined three natural products was optimized based on the acute paw swelling mouse model with a Uniform Design methodology. The therapeutic effect of TRC combination on chronic inflammation was investigated by using the collagen-induced arthritis (CIA) rat model based upon the following indexes: the volume of paw swelling, arthritis score, serum mediators and histological examination as well as immunohistochemical staining. The levels of alanine aminotransferase (ALT) and aspartate transaminase (AST) in serum were measured and the pathological sections of liver and kidney were analysed. LD_50_ was measured based on the acute oral toxicity (AOT) standard method.

**Results:**

The best formulation was the three components combined at the same mass proportion revealed by the Uniform Design methodology. This combination could significantly reduce the paw swelling in acute paw swelling mouse model, could reduce paw swelling and alleviate the damage in joint structural of ankle, cartilages and fibrous tissue in CIA rat model. The dose relationship was clear in both cases. Immunohistochemical staining of ankle tissue revealed that TRC combination was able to inhibit the expression of NF-κB p65 and TNF-α which were closely related to the inflammatory process. Analysis of serum mediators revealed TRC combination could inhibit the production of TNF-α, IL-1β, and IL-6 in the serum. Toxic study revealed this formulation was low toxic, LD_50_ was larger than 5 g/kg, both the level of ALT and AST and histopathology in the liver and kidney exhibited no distinctions between the TRC combination and the blank group, no mortality occurred at the administered doses of 5 g/kg.

**Conclusions:**

The results showed this formulation could provide a novel potent treatment for acute and chronic inflammation (RA) without side effect like gastric injury occurring in NSAIDs.

## Background

Inflammation is a protective mechanism against different deleterious stimuli, such as tissue damage, microbial invasion, and chemical exposure [1]. Acute inflammation response is an immune and defensive function to prevent and control the invasion of pathogens, which manifests mainly as lymphocyte infiltration and activation. While chronic inflammation is caused by the pro-inflammatory mediators persistently invading cells and tissues, which manifests as a variety of diseases such as rheumatoid arthritis, cardiovascular and cerebrovascular diseases [2].

RA is a complex multisystem chronic inflammation with characteristics of synovial hyperplasia and progressive destruction of bone and cartilage. The major symptoms are pain, joint swelling and exercise difficulties. The incidence of RA is about 1% of the population all over the world, mainly concentrating in the age above 40 years old [3]. Since the pathogenic process of RA is complicated and the mechanism is not understood clearly, there is no effective method or drug treatment for RA at present. Over the past years, nonsteroidal anti-inflammatory drugs (NSAIDs) and disease-modifying anti-rheumatic drugs (DMARDs) as the major treatment medicines have been widely used to control and delay the aggravations of RA, but now these treatments have been clinically restricted because of its adverse effect in gastrointestinal and other organs.

A growing number of anti-inflammatory targets are revealed recently [4], including COX-1, COX-2, cytokines and their receptors, nuclear factor (NF)-κB, mitogen-activated protein kinase and so on. The number of inflammatory mediators and pathways are so large that single drug is impossible to cure or prevent RA. Combination of the multi-target drugs has become one major strategy for the treatment of RA and a large number of studies and clinical researches have done in this way. For example, TMP and methotrexate combination have the therapeutic effect for arthritis clinically and alleviate the side effect of leflunomide [5]. Sinomenine and methotrexate combination in clinical can improve the treatment effect for RA and reduce the common dose of single drug. Traditional Chinese prescriptions Guizhi decoction and methotrexate combination also show good treatments for RA [6]. Natural flavonoids usually exhibit multi-target function; some of them show significant anti-inflammatory activities and minimal side effect in treatment of rheumatoid arthritis [7]. Among them, curcumin (Cur), a yellow compound derived from the plant *Curcuma longa Linn*, and resveratrol (Res), a white powder found in grapes, red wine, are the most recognized anti-inflammatory drugs. Curcumin can mitigate inflammatory responses of macrophages stimulated by lipopolysaccharide and inhibit a variety of inflammation markers, such as cyclooxygenase, lipoxygenase and nitric oxide synthase in different extent [8]. Resveratrol has potent anti-inflammatory effect via kinds of inflammatory pathways in vitro *and* in vivo [9]. These two components can increase the multiple immunosuppressive activities in cell proliferation and antibody production; can mediate the anti-inflammatory effect by targeting the similar signalling pathway. Tetramethylpyrazine (TMP), a major active component obtained from *Ligusticum wallichi Franchat* (chuanxiong), has been used to treat cardiovascular and inflammatory diseases clinically in China for a long time [10]. TMP and tanshinol IIA combination show synergistic or additive effect in protecting the neuron against hypoxia/ischemia both in vitro *and* in vivo [11]. TMP has angiogenesis and vessel protection effect and can protect ischemic brain from injury in rats by suppressing inflammatory reaction. In addition, TMP can protect articular cartilage and chondrocytes from deterioration and apoptosis in rabbits [12]. Different symptoms of inflammation may have a common pathogenesis, so the treat strategy of TMP combined with natural flavonoids is possible to enhance the synergetic effect in anti-inflammatory or anti-arthritic. Up to now, the combined effect of TMP and flavonoids on acute and chronic inflammation have not been reported before. In this paper, the therapeutic effect of this combination was investigated using the carrageenan-induced acute paw edema inflammation mouse model and the CIA rat model.

## Methods

### Materials

Tetramethylpyrazine, curcumin, resveratrol (98%) were purchased from Aladdin (Shanghai, China). All the other reagents were analytical grade.

### HPLC of the TRC combination

**Fig. 1:**
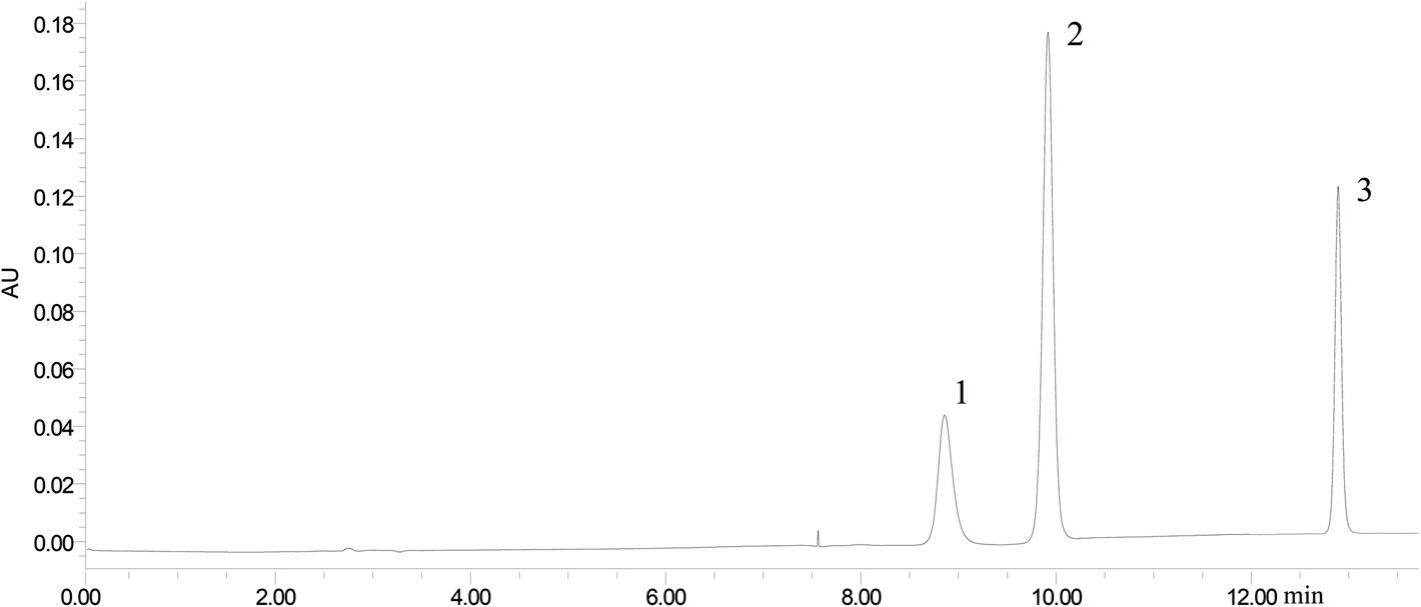
The HPLC of Curcumin, Resvertrol and TMP. The HPLC chromatogram of standard substances monitored at 254 nm. Peak 1, 2, 3 are TMP, Resvertrol, and Curcumin, respectively

HPLC of the TRC combination was demonstrated on Waters 2695 Alliance instrument (Agilent, German), with Kromasil C_18_ column (5 μm, 4.6 mm × 250 mm) as column, Methanol (A) and water containing 0.5% acetic acid (B) as the mobile phase, the column temperature was set at 35 °C. The detection wavelength was set at 254 nm. A multistep gradient program was set as follows: 45–50% A at 0–6 min, 50–95% A at 6–9 min, and 95–45% A at 9–14 min. The flow rate was kept at 1 mL/min. Tetramethylpyrazine, curcumin and resveratrol as index components were examined. HPLC analysis was performed in triplicate. A typical chromatogram was shown in Fig. 1.

### Acute inflammation mouse model and treatment

Adult male Kunming mice (body weight range 20 ± 2 g) purchased from Beijing HFK Bioscience Co., LTD (production license No.: SCXK 2014–0004) were used only once. All mice were housed at a constant climate 21 ~ 25 °C and relative humidity 40 ~ 60% with a 12 h light/dark cycle. They adjusted to the environment for seven days before the experiment and were free access to food and water. No side effects were observed in any of the studied animal groups.

In order to obtain the satisfied prescription, the dose range for each component was rationalized by the Uniform Design 3.0 software (China). The dose range was designed based upon its single practical dosage reported in the literature [13, 14]. The LD_50_ for each component was more than 800 mg/kg via the oral route in mice. So in the following optimization process, each component varied in six different doses levels according to the rule of equal proportion or equal difference (Table 1), and an U(3^6^) factorial design was implemented (Table 1). Considering the common doses of nature products were not more than 200 mg/kg in mice, so here 25, 50, 75, 100, 125 and 150 mg/kg were chosen as the six different dosages for each component. The optimal dosages for each component could be analysed easily and accurately based upon the software. Mice were randomly divided into eight groups, the blank group was oral administrated with normal saline and the control and the other six experimental groups were oral administrated with the designed groups (listed in Table 2) for 7 days. After the last administration, in control group and experimental groups, λ-carrageenan (sigma, 22,049-5G-F) (50 μL, 1% *w*/*v*) solution in 0.9% saline was subsequently injected into the right hind-paw, while the blank group was treated with the 0.9% saline only. The difference in paw edema thickness between the right and left foot was measured with vernier calipers at 1, 2, 3 and 4 h after injection. The results of inhibition proportion were summarized and obtained from each experimental animal.

**Table 1:** Uniform Design table (3 factors, 6 levels)

Variables	Symbol	1	2	3	4	5	6
TMP (mg/kg)	X1	25	50	75	100	125	150
Res (mg/kg)	X2	25	50	75	100	125	150
Cur (mg/kg)	X3	25	50	75	100	125	150

**Table 2:** Experimental design of the U3^6^and its corresponding efficiency

Group	TMP	Res	Cur	Efficiency (%)
1	25	50	75	10.35 ± 1.48
2	50	100	150	14.31 ± 1.77
3	75	150	50	20.41 ± 1.39
4	100	25	125	12.23 ± 1.71
5	125	75	25	10.01 ± 2.02
6	150	125	100	20.27 ± 1.21

### Dose verification

To verify the accuracy of the screening dose, another acute inflammation mouse model was established and the same treatment as the dose screening was done, the optimized prescription was administrated to the model mice for 7 days, the inhibition effect of the optimized dose in acute inflammation model was investigated, the results was shown in Table 3.

**Table 3:** Predicted and experimental efficiency of the optimal formulation on acute inflammatory mice model

Group	TMP (mg/kg)	Res (mg/kg)	Cur (mg/kg)	Experimental Efficiency (%)	Predicted Efficiency (%)
1	150	150	150	25.98 ± 2.68	26.17

**Table 4:** The three dose group of TRC combination on CIA rats model

Group	TMP(mg/kg)	Res (mg/kg)	Cur (mg/kg)
Low dose	25	25	25
Middle dose	50	50	50
High dose	75	75	75

**Table 5:** The concentration of ALT and AST

	The blank group	The model group	The positive group	The TRC combination		
				25 mg/kg	50 mg/kg	75 mg/kg
ALT(U/L)	27.88 ± 1.48	44.46 ± 2.78	33.77 ± 2.37	33.68 ± 1.75	30.56 ± 2.70	31.77 ± 1.77*
AST(U/L)	105.50 ± 1.72	120.61 ± 1.97	105.89 ± 1.28	125.01 ± 3.41	117.79 ± 3.32	105.89 ± 2.21*

Data are expressed as Mean ± SEM and analysed by one-way ANOVA for each parameter separately. As compared to the blank group, the model group have a significant (*P* < 0.05). As compared to the model group, the positive and high dose group have a significant (*P* < 0.05). The TRC combination was administered orally daily for 26 days

### CIA rats model and drug administration

CIA rats model was built with 36 female Sprague-Dawley(SD) rats (140 to 160 g), which were purchased from the Beijing HFK Bioscience Co., LTD (production license No.: SCXK 2014–0004). Rats were used once only. All animals were housed as the above descriptions in acute inflammation mouse model.

CIA model was built as described in previous paper [15]. In brief, bovine type II collagen (BII; Chondrex, USA) in 0.1 M acetic acid mixed in an equal volume of complete Freund’s Adjuvant(CFA; Chondrex, USA). Then the mixture emulsified completely and subcutaneous injected at the base of the tail in a volume of 200 μL on day 1. Additionally, six rats were injected with 200 μL saline as the blank group. On day 7, another new BII mixed CFA was injected in half volume with the same method as described above. On day 10, the model rats were randomly divided into five experimental groups with six rats in one group. The blank and model group were oral administrated intragastrically with normal saline; the other four groups were given three different dosages of TRC combination (Table 4) every day and positive drug (methotrexate, MTX, 7.8 mg/kg) twice a week until they were sacrificed on day 36. The dosages of administration were half amount converted by mouse. The following assessment results obtained from each experimental animal.

### Assessment of arthritis progression

The hind paw volume of all rats were measured by plethysmometer (YLS-7B, Shanghai, China) on day 10, 14, 16, 18, 20, 24, 28, 32 and the progression of CIA was evaluated by macroscopic scoring of paws. Arthritis scores of the rats were evaluated visually by two independent observers. The following criteria were used to score arthritis: 0, no swelling; 1, redness or swelling or both in one joint; 2, redness or swelling or both in more than one joint; 3, redness or swelling or both in the entire paw; 4, deformity or both. The maximum score of single paw was 4 and a single rat was 8 [16].

### Assessment of inflammatory mediators

To measure pro-inflammatory cytokines TNF-α, IL-6 and IL-1β levels, all rats were sacrificed and blood were collected in centrifuge tubes on day 36 of the experiment. The plasma samples were allowed to stand for half an hour and then centrifuged at 3000 rpm/min for 15 min at room temperature. The clear serum were collected and placed in a refrigerator at −80 °C, which were measured by enzyme-linked immunosorbent assay (ELISA) kits (eBioscience, USA) for estimation of cytokine levels.

### Histopathological analysis and immunohistochemistry

On day 36, the ankle joints were harvested and fixed in 4% paraformaldehyde for 3 days. Then the samples were decalcified in 10% eathylene diamine tetraacetic acid (EDTA) of PBS solution and changed once a week for 42 days at 25 °C. The ankle joints became soft; after dehydration they were buried into paraffin bulky and then cut into 5 μm thick slices, which were stained with hematoxylin and eosin. The pathological states of cartilage degradation and bone destruction were analysed on the basis of cartilage, synoviocytes and fibrous tissue. All scores were based on a scale of 0–5 according to the following criteria: 0, normal, 1, minimal, 2, mild, 3, moderate, 4, marked, and 5, severe, respectively [17].

The immunohistical analysis of NF-κB p65 and TNF-α were carried out according to the following procedure. Briefly, paraffin sections of ankle joints were dewaxed, hydrated and then incubated for 5 min with 3% H_2_O_2_. Normal rabbit serum was incubated for 30 min at room temperature and mouse monoclonal antibodies of NF-κB p65 and TNF-α (Proteintech Group, USA) were added at dilution of 1:200 overnight at 4 °C according to the manufacturer’s instructions. Then second antibody was incubated and visualized with 3, 3-diaminobenzidine. The final sections were counterstained with haematoxylin. As a control, PBS was used instead of the primary antibody.

### AST and ALT concentration and histological assessment of liver and kidney

The clear serum were collected on day 36 and stored at −80 °C before analysed. The level of AST and ALT in serum, two indexes reflected the toxics, was analysed with the Biological kit purchased from Nanjing Jiancheng Bioengineering Institute (Nanjing, China). Liver and kidney tissue were excised and fixed in 4% paraformaldehyde. They were buried into paraffin and cut into 5 μm thick slices, then stained with haematoxylin-eosin. The pathological states of each section was observed and identified at ×100 magnification under a Nikon light microscope.

### Oral acute toxicity (AOT) of TRC combination in mice

The AOT dose, 0.5 g/kg, 1.08 g/kg, 2.32 g/kg, 5 g/kg, was designed according to the Horn’s method [18]. The Kunming mice (6 mice/sex/group) were randomly divided into five groups. After fasting for 12 h, each group was oral administrated 0.5 g/kg, 1.08 g/kg, 2.32 g/kg, 5 g/kg and the vehicle control(10 mL/kg, distilled water) respectively only once. After administration, 4 hours of continuous observation was carried out, and then in the following 14 days, the appearance behaviour, poisoning symptoms and death were observed in the morning and afternoon. On the last day all the mice were sacrificed and the organs were harvested, compared the ensemble indexes and weighed.

### Statistical analysis

All data were processed by authorized SPSS 17.0 software. Date was analysed using one-way ANOVA followed by Dunnett’s t-test to assess differences between the study groups. Differences were considered statistically significant as P was less than 0.05.

## Results

### Dose optimization in the TRC combination

λ-carrageenan as an inflammatory agent can repeatability induce mouse paw edema inflammation model with obvious pathological features. In order to investigate optimal combination with good compatibility based on their common dosage in mouse experiment, the dose value for each component was chosen and designed according to the rule of same equal proportion or equal difference, so 3 factors and 6 levels were designed based upon Uniform Design U (3^6^). The single contributed factor (TMP (X1), Res (X2), and Cur (X3)) for the ensemble inhibitory rate (Y) was extracted as a second-order polynomial equation: Y = 7.42 + 5.02*10^−4^*X2^2^ + 3.31*10^−4^ *X1*X3. The results were shown in Table 2. The ANOVA of the quadratic regression model showed that this model was highly significant (*p* = 0.0055). Besides, the determination coefficient (R^2^) of the regression model was 0.9843, indicated a close correlation among them. The *p* value of X2^2^ and X1*X3 were 0.009 and 0.0203 respectively, indicating that the interaction between TMP and Cur could increase the entire efficiency. Therefore, the simulation results indicated that with every component consisting of TMP (X1), Res (X2) and Cur (X3), all in the same dose of 150 mg/kg, the largest inhibition effect about 26.17% could be achieved. It also concluded that the above formulation was the most potent formulation.

### Validation of the model equation

According to the above model equation, as 150 mg/kg of TMP, 150 mg/kg of Res, and150 mg/kg of Cur combined together, the most potent formulation was predicted with the inhibitory effect of 26.17%. In order to verify the accuracy of the model, the formulation with 150 mg/kg of TMP, 150 mg/kg of Res and 150 mg/kg of Cur was combined together and its anti-inflammatory efficacy in acute inflammation model was implemented. The experiment result was listed in Table 3. A mean value of 25.98 ± 2.68% was obtained, which was very close to the data predicted by model, 26.17%. So in this combination, TMP, Res and Cur could increase the ensemble effect, and as they had the same mass proportion in the formulation, the dosage had the most potent efficiency. The experimental result matched very well with the model predicted, indicating that the model was accurate.

### Effect of TRC combination on arthritis in CIA rats

**Fig. 2:**
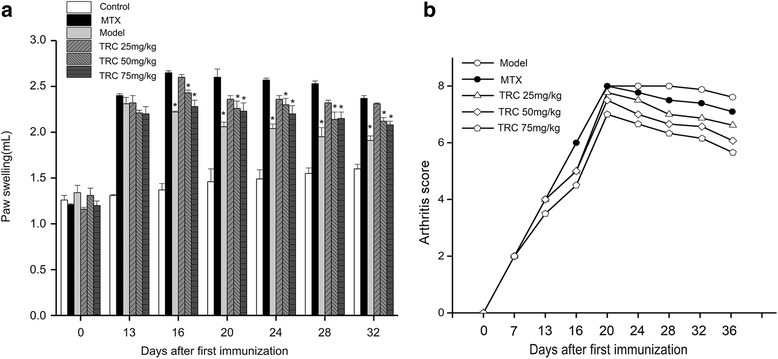
Effect of TRC combination on the progression of arthritis in CIA rats. Rats were orally administered TRC combination from day 13 to 36. The volume of paw swelling (**a**) and arthritis score (**b**) were evaluated. The hind paw volume of every rat in different group was measured twice a week

**Fig. 3:**
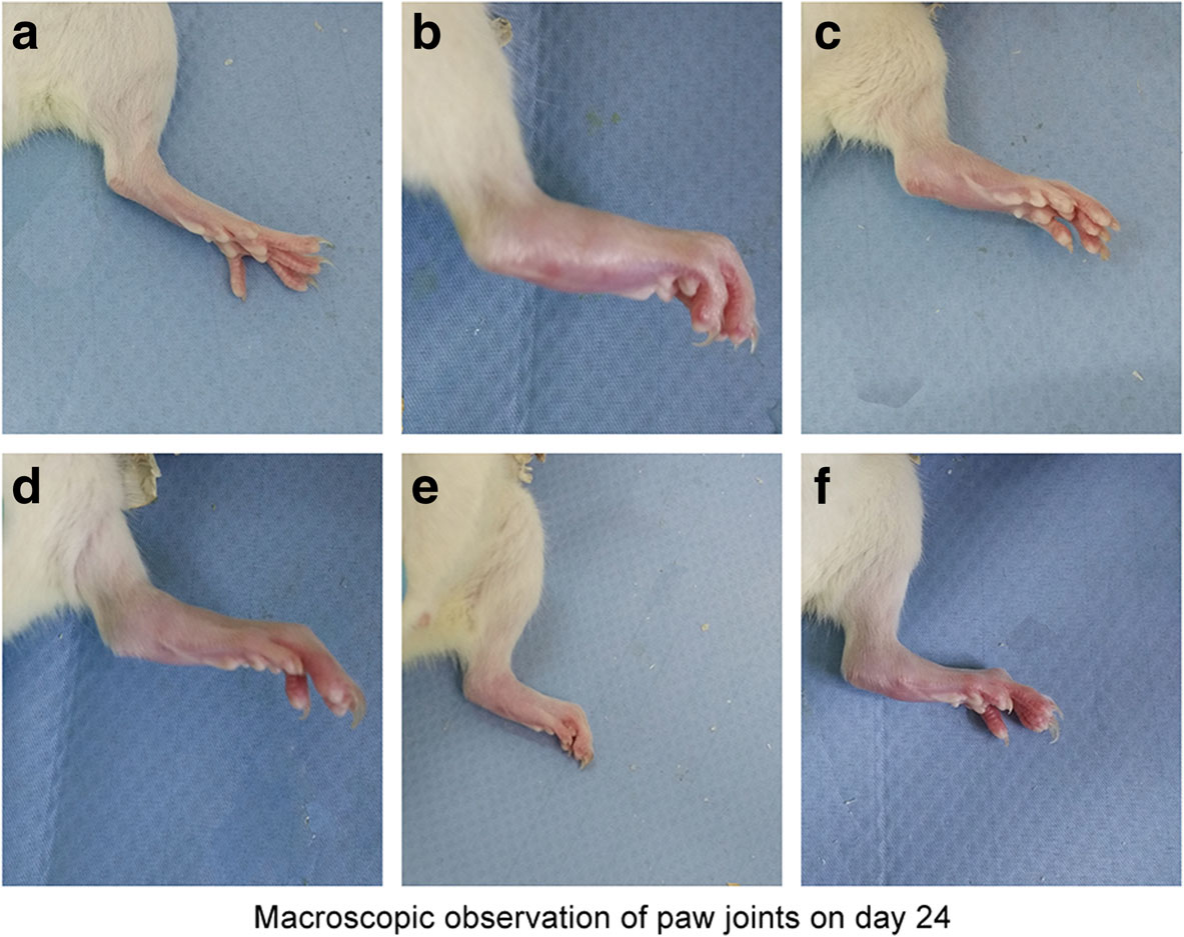
Macroscopic signs of arthritis on day 28. Macroscopic signs of arthritis included swelling, redness and deformity in ankle joints. **a** Normal; (**b**) Model; (**c**) MTX; the TRC combination (**d**) 25 mg/kg; (**e**) 50 mg/kg; (**f**) 75 mg/kg. Compared with Normal (**a**), obvious arthritis symptoms of erythema and edema were observed in Model (**b**). Different dose (**d**, **e**, and **f**) could relieve the arthritis symptoms and the high dose could significant attenuated edema as MTX

In this experiment, the therapeutic effect of TRC combination to CIA rats was evaluated. After the primary immunization, the rats passed through a series of arthritic symptoms progressively. From day 10, the occurrence of arthritis was obvious. The paws of rats gradually swelled and could not move freely in model group. The treated group were oral administrated TRC combination (25, 50 and 75 mg/kg) from day 13, respectively. After oral administration of TRC combination for 4 weeks, the treated groups showed a decrease in arthritic paw swelling and arthritis scores, and recovery of movement capacity in CIA rats (Fig. 2). At the end of the experiment, the swelling rate decreased from 66.42% to 30% in the high dose group. In contrast, the model group showed severe symptoms of arthritis (Fig. 3). The overall therapeutic effect was dose-dependent; the swelling rate of rats’ feet was sufficient to be comparable to that of the positive group administrated MTX.

### Effect of TRC combination on inflammatory mediators in CIA rats

**Fig. 4:**
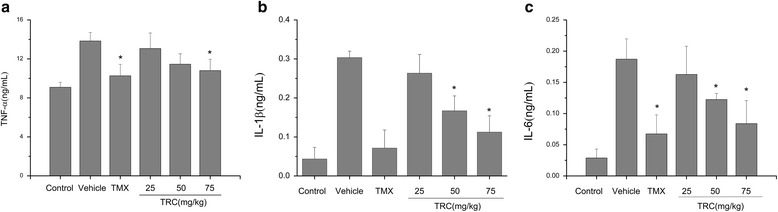
Effect of TRC combination on inflammatory mediators in serum of CIA rats. **a** The concentration of TNF-α; (**b**) The concentration of IL-1β; (**c**) The concentration of IL-6

TNF-α, IL-1β and IL-6 as pro-inflammatory cytokines possessed a multitude of biological activities associated with the immunopathology of RA. Their levels in serum were analysed via Multiplex immunoassays, shown in Fig. 4. Treated with TRC combination, the level of TNF-α, IL-1β and IL-6 was significantly decreased in serum, and this trend was in a concentration-dependent manner compared with in model group (*P* < 0.05).

### The histological changes and immunohistochemical staining of TRC

**Fig. 5:**
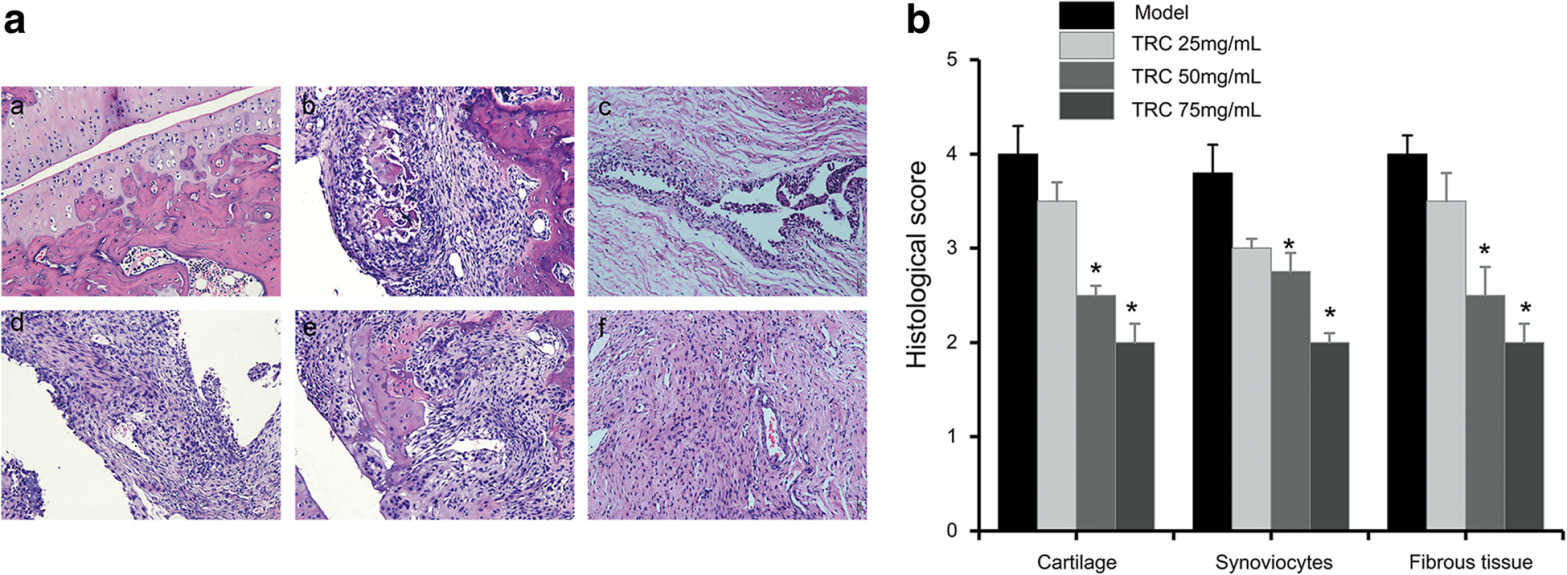
Haematoxylin and eosin staining of the ankle joints in CIA rats. **a** HE (100×). **b** The histological score of cartilage damage, synoviocytes, fibrous tissue in ankle joint of CIA rats

Histochemically analysis of synovial and articular cavities of ankle joints from CIA rats was conducted. A typical characteristic for rheumatoid arthritis was observed in model group: structural damage of ankle, widely damaged cartilages, intense inflammatory cell infiltration and the proliferation of fibrous tissue (Fig. 4a (b)). While in the blank group, normal ankle joint characteristics was observed: clear, healthy joint space and tissues. Four weeks administration of TRC combination resulted in a decrease in the inflammatory cell infiltration, less thickness of the synovium and less volumetric increase in the synovial space (Fig. 5a). Especially, oral administration of high dose of TCR combination (75 mg/kg) gave a better histopathological assessment significantly. At the end of experiment, the deterioration of arthritis was reduced a lot according to the histopathological scores (Fig. 5b).

**Fig. 6:**
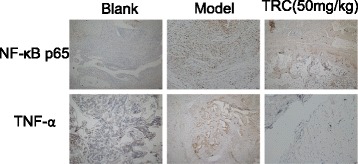
Immunohistochemical analysis of NF-κB p65 and TNF-α in the ankle joints of CIA rats (100×). At the end of the experiment, paraffin sections of ankle joints were used to immunohistochemical research to show the presence of NF-κB p65 and TNF-α. Compared with the blank group, the model group displayed increase expression. On the other hand, the 50 mg/kg dose of the TRC combination showed weak cytoplasmic staining

NF-κB signalling pathways play an important role in the regulation of inflammatory mediator production and TNF-α is the key mediator. For CIA rat groups treated by the high dose of TRC combination (50 mg/kg), weak stain for NF-κB p65 and TNF-α suggested their express was regulated low down, so the TRC combination could deactivate or decrease both phosphor-p65 and TNF-α shown in Fig. 6. On the contrast, the control model showed heavily staining.

### The level of AST and ALT and histopathology in rats

**Fig. 7:**
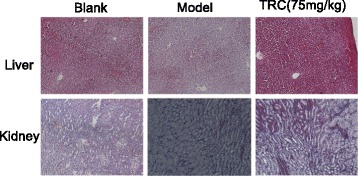
Haematoxylin and eosin staining of kidney and liver tissue in rats. Histological findings of kidney and liver tissue in different rat groups were conducted during the CIA rats’ experiments. Photomicrographs were chosen from different groups at 100 ×

Compared with the blank control group, the level of AST/ALT in model group was significantly higher (Table 5), and it lowered in the TRC treatment group, especially in the high dose group with statistical significance. At the three doses of the TRC combination, no structural change in histopathology was found (Fig. 7). The liver tissue organization of the rats in every group is normal and the hepatic lobules of them are well-arranged. In the kidney, the structure of renal cortex, tubular interstitium are integrity. So the TRC combination didn’t express any toxic effects to liver and kidney.

**Table 6:** food and water intake (AOT study)

Parameter	Female					Male				
	Control	TRC combination(g/kg, oral)				Control	TRC combination(g/kg, oral)			
		0.5	1.08	2.32	5		0.5	1.08	2.32	5
Mean food intake (g)	5.5 ± 0.04	5.62 ± 0.02	6.58 ± 0.03	6.33 ± 0.04	6.83 ± 0.05	7.33 ± 0.04	7.83 ± 0.01	7.66 ± 0.04	7.33 ± 0.02	7.83 ± 0.02
Mean water intake (mL)	12.33 ± 0.02	12.66 ± 0.01	12.83 ± 0.01	11.33 ± 0.02	11.66 ± 0.02	11.16 ± 0.03	13.75 ± 0.03	14 ± 0.02	14.33 ± 0.03	13.66 ± 0.04

Data was expressed as mean ± SEM and analysed by one-way ANOVA. Compared to the control, the food and water intake of TRC combination don’t express any difference

### AOT study of TRC combination in mice

The toxicity assay was conducted according to the Horn’s method. Mice were treated with four different doses of TRC combination, 0.5 g/kg, 1.08 g/kg, 2.32 g/kg, 5 g/kg, respectively once a time, there were no mortality until day 14. The treated male and female mice showed the same increase in food intake and water intake compared to the control mice (Table 6). The body weight and the relative organ weights for the treated group had no significant increase (*p* > 0.05) compared to the control group on day 14 (Table 7). The acute toxicity data indicated that the calculated LD_50_ value for the TRC combination was more than 5 g/kg.

**Table 7:** The relative organ weights (AOT study)

Parameter	Female					Male				
	Control	TRC combination(g/kg, oral)				Control	TRC combination(g/kg, oral)			
		0.5	1.08	2.32	5		0.5	1.08	2.32	5
Terminal body	32.5 ± 1.24	31.40 ± 1.74	34.33 ± 1.53	31 ± 1.64	30.33 ± 0.57	39.25 ± 2.64	38.60 ± 2.40	39.00 ± 2.64	38.00 ± 2.35	39.67 ± 1.53
Brain(%)	1.26 ± 0.16	1.32 ± 0.20	1.24 ± 0.23	1.60 ± 013	1.47 ± 0.08	1.15 ± 0.20	1.32 ± 0.20	1.09 ± 0.17	5.93 ± 0.08	1.15 ± 0.06
Heart(%)	0.54 ± 0.04	0.54 ± 0.07	0.53 ± 0.06	0.56 ± 0.07	0.54 ± 0.02	0. 58 ± 0.05	0.59 ± 0.01	0.59 ± 0.08	0.58 ± 0.02	0.59 ± 0.08
Lung(%)	0.88 ± 0.12	0.68 ± 0.06	1.04 ± 0.45	0.86 ± 0.17	0.75 ± 0.05	0.68 ± 0.04	0.78 ± 0.10	0.70 ± 0.05	0.67 ± 0.06	0.75 ± 0.05
Liver(%)	5.12 ± 0.16	5.06 ± 0.14	5.11 ± 0.29	5.55 ± 0.63	4.97 ± 0.21	5.46 ± 0.14	5.60 ± 0.06	5.64 ± 0.12	5.74 ± 0.03	5.59 ± 0.20
Kidney(%)	1.24 ± 0.06	1.26 ± 0.10	1.21 ± 0.06	1.34 ± 0.13	1.22 ± 0.02	1.64 ± 0.19	1.56 ± 0.21	1.60 ± 0.23	1.68 ± 0.14	1.54 ± 0.16
Spleen(%)	0.39 ± 0.03	0.37 ± 0.01	0.39 ± 0.03	0.39 ± 0.05	0.39 ± 0.05	0.37 ± 0.03	0.37 ± 0.10	0.38 ± 0.03	0.35 ± 0.08	0.37 ± 0.04
testis	-	-	-	-	-	0.51 ± 0.04	0.55 ± 0.06	0.55 ± 0.06	0.51 ± 0.05	0.51 ± 0.08

Data was expressed as mean ± SEM and analysed by one-way ANOVA. Compared to the control, the relative organ weights of TRC combination don’t express any difference

## Discussion

Natural products with some advantages like potent anti-inflammatory effect, low toxicities, as well as rich sources, receive great attention in recent years [19]. Inflammation disorders usually involve many complicated mechanisms and pathways, so one drug was weak in treating inflammation. Therefore, combination of drugs becomes a practical approach. Natural products usually have multiple targets interactions and show high therapeutic effect, so combinations of natural products are expected to be useful in the treatment of acute and chronic inflammatory diseases in practice [20]. In this paper, TMP and two typical flavonoids (Resvertrol, Curcumin) are combined with consideration of adding their individual anti-inflammatory effect and different targeting effect; thus, a synergistic antiflammatory effect has been desired. The screening method is conducted based on an acute inflammatory model, and the optimized combination was verified in CIA model.

The mass proportions of each component in the formulation were optimized based on Uniform Design Methodology. The representative test points and multiple stepwise regression analysing method can reduce the number of experiments greatly [21]. At the same time, the optimal experimental parameters could be predicted by theoretical analysis. In this paper, when the mass proportion was the same for each component in the combination, the inhibition on paw swelling of mouse were most effective and their efficacy was dose-dependent. In acute inflammatory mouse model, after 1 week administration of TRC combination, the paw edema swelling of mice was obviously decreased. The arthritis score was also lower than the model group. This combination was verified in the chronic inflammation rate model, built by injection of CII to the rat inducing paw edema, synovial hyperplasia, and cartilage erosion. After administration of the optimized TRC formulation with three doses for 4 weeks, a good treatment effect was revealed from direct symptom and tissue staining. And histological analysis indicated a significant difference between the treated group and the model group. Obvious reduction was observed in synovial hyperplasia, pannus formation, and cartilage damage and bone erosion respectively. Compared with single TMP or flavones which have been documented in the literature [22, 23], the TRC combination displays better therapeutic effect to arthritis.

The pro-inflammatory cytokines like TNF-α and IL-1β could induce the expression of inducible nitric oxide synthase and cyclooxygenase-2 in macrophages and synoviocytes, resulting in the marked elevation of NO and PGE_2_ in synovial fluid or serum from RA patients [24, 25]. Higher concentration of TNF-α and IL-1β were detected in serum and synovial fluid of RA rats’ model, they were low down after administration of the TRC combination. This indicated that TRC formulation could inhibit the secretion of TNF-α, IL-1β and IL-6 generally and this function was dose dependent. NF-κB plays a pivotal role in inflammation through the transcription of genes encoding pro-inflammatory cytokines, adhesion molecules, and chemokines [26]. Immunohistochemical staining indicated that NF-κB activity was significantly increased in CIA rats synovium, while was suppressed heavily by TRC combination. Inhibition of NF-κB can interpret why the expression of many inflammatory cytokines was suppressed as revealed above.

The level of ALT and AST, the histopathology of liver and kidney, all of these toxicity indexes exhibited no remarkable changes upon 30 days oral administration of the TRC combination at the dose of 75 mg/kg . AOT studies determined the LD_50_ values for the TRC combination was more than 5 g/kg, at this dosage there were no mortality (0%) within 14 days. The mice indexes, such as body weight, organ weight, are normal compared with the control group. So this formulation was low toxic at present. The conclusion for it non-toxic needs more toxicity test such as repeated dose 28-day (sub-acute) oral toxicity to ensure the security of the combination.

In conclusion, the therapeutic effect of TRC combination on CIA model has been investigated significantly. The optimal combination was that tetramethylpyrazine, resveratrol and curcumin (TRC) combined in equal mass proportions. This combination could obviously reduce arthritis symptoms like swelling of foot, structural damage of ankle, widely damaged cartilage, intense inflammatory cell infiltration and the proliferation of fibrous tissue. These therapeutic effect partially resulted from the direct suppression of the production of pro-inflammatory cytokines and mediators in the joints and the circulating blood, which partially contributes inactivation of NF-κB signalling pathways. The results showed this formulation could provide a novel potent treatment for acute and chronic inflammation (RA) without side effect like gastric injury occurring in NSAIDs. These results highlight the potential therapeutic effect of TRC combination on RA, and require further study.

## References

[1] Gautam R, Jachak SM (2009). Recent developments in anti-inflammatory natural products. Med Res Rev.

[2] Don D, Sin MSP, Marciniuk D (2006). Can inhaled fluticasone alone or in combination with salmeterol reduce systemic inflammation in chronic obstructive pulmonary disease? Study protocol for a randomized controlled trial. BMC Pulm. Med.

[3] Bingham CO (2002). The pathogenesis of rheumatoid arthritis: pivotal cytokines involved in bone degradation and inflammation. J Rheumatol.

[4] Grivennikov SI (2010). Immunity, Inflammation, and Cancer. Cell.

[5] Maione P, Gridelli C, Troiani T (2006). Combining Targeted Therapies and Drugs with Multiple Targets in the Treatment of NSCLC. Oncologist.

[6] Sun Y, Yao Y, Ding CZ (2014). A combination of Sinomenine and Methotrexate reduces joint damage of collagen induced arthritis in rats by modulating osteoclast-related cytokines. Int Immunopharmacol.

[7] Wang Q, Kuang H, Su Y (2012). Naturally derived anti-inflammatory compounds from Chinese medicinal plants. J Ethnopharmacol.

[8] Lü S, Wang Q, Li G, Sun S, Guo Y, Kuang H (2015). The treatment of rheumatoid arthritis using Chinese medicinal plants: From pharmacology to potential molecular mechanisms. J Ethnopharmacol.

[9] Bisht K, Wagner KH, Bulmer AC (2010). Curcumin, resveratrol and flavonoids as anti-inflammatory, cyto- and DNA-protective dietary compounds. Toxicology.

[10] Liu HT, Du YG, He JL (2010). Tetramethylpyrazine inhibits production of nitric oxide and inducible nitric oxide synthase in lipopolysaccharide-induced N9 microglial cells through blockade of MAPK and PI3K/Akt signaling pathways, and suppression of intracellular reactive oxygen species. J Ethnopharmacol.

[11] Tang Q, Han R, Xiao H (2012). Neuroprotective effect of tanshinone IIA and/or tetramethylpyrazine in cerebral ischemic injury in vivo and in vitro. Brain Res.

[12] Ju XD, Deng M, Ao YF (2010). The protective effect of tetramethylpyrazine on cartilage explants and chondrocytes. J Ethnopharmacol.

[13] Highab S, Muhammad D, Aliyu M. Effect of resveratrol on some biochemical parameters in lead-intoxicated male wistar rats. 2016;5(3):1-11.

[14] Adewunmi CO, Odebiyi OO (2008). Schistosomicidal Activity of Tetramethylpyrazine from Hook. Stem Bark. Int J Crude Drug Res.

[15] Kong X (2013). The suppressive effect of Saposhnikovia divaricata (Fangfeng) chromone extract on rheumatoid arthritis via inhibition of nuclear factor-κB and mitogen activated proteinkinases activation on collagen-induced arthritis model. J Ethnopharmacol.

[16] Schett G, Tohidast-Akrad M, Smolen JS (2000). Activation, differential localization, and regulation of the stress-activated protein kinases, extracellular signalregulated kinase, c-JUN N-terminal kinase, and p38 mitogen-activated protein kinase in synovial tissue and cells in rheumatoid arthritis. Arthritis Rheum.

[17] Bendele A, McAbee T, Sennello G (1999). Efficacy of sustained blood levels of interleukin-1 receptor antagonist in animal models of arthritis: comparison of efficacy in animal models with human clinical data. Arthritis Rheum.

[18] Kandhare AD, Bodhankar SL, Mohan V (2016). Acute and repeated doses (28 days) oral toxicity study of Vicenin-1, a flavonoid glycoside isolated from fenugreek seeds in laboratory mice. Regul. Toxicol. Pharmacol.

[19] Serumfini M, Peluso I, Raguzzini A (2010). Flavonoids as anti-inflammatory agents. Proc Nutr Soc.

[20] Chen S (2011). Natural products triggering biological targets--a review of the anti-inflammatory phytochemicals targeting the arachidonic acid pathway in allergy asthma and rheumatoid arthritis. Curr Drug Targets.

[21] Guo D. Uniform design method and its application. J Math Med. 2005;18(1):69–71.

[22] Mu CX, Liu GL, Tian H (2014). Effect of tetramethylpyrazine on serum levels of IL-1beta, IL-6, and IL-2, and NO and PGE_2_ in the synovial fluid of CIA rats: an experimental research. Zhongguo Zhong Xi Yi Jie He Za Zhi.

[23] Nishizaki T, Kanno T. Resveratrol: A Candidate Drug for Treating Rheumatoid Arthritis. Rheumatoid Arthritis-Treatment.In Tech. 2012;269–84.

[24] Chen Y, Yang L, Lee TJ (2000). Oroxylin A inhibition of lipopolysaccharide-induced iNOS and COX-2 gene expression via suppression of nuclear factor-κB activation. Biochem Pharmacol.

[25] Posadas I, Terencio MC, Guillén I (2000). Co-regulation between COX-2 and iNOS expression in the time-course of murine inflammation. Archiv Für Experimentelle Pathologie Und Pharmakologie.

[26] Simmonds RE, Foxwell BM (2008). Signalling, inflammation and arthritis NF-κB and its relevance to arthritis and inflammation. Rheumatology.

